# Meta-Analysis of Capecitabine versus 5-Fluorouracil in Advanced Gastric Cancer

**DOI:** 10.1155/2023/4946642

**Published:** 2023-06-27

**Authors:** Zhongliang Wu, Xingfa Zhang, Chongxiang Zhang, Yi Lin

**Affiliations:** Department of Gastrointestinal Surgery, The Third Affiliated Hospital of Guizhou Medical University, Duyun, Guizhou Province, China

## Abstract

**Objective:**

To investigate the effect of capecitabine versus 5-fluorouracil in advanced gastric cancer patients.

**Methods:**

We searched PubMed, Cochrane Library, Embase, and other databases from database establishment to June 2022, containing randomized controlled trials (RCT) on capecitabine and 5-fluorouracil in advanced gastric cancer patients. A meta-analysis was conducted to evaluate the effect of capecitabine versus 5-fluorouracil on overall response rate, neutropenia, thrombocytopenia, stomatitis, hand-foot syndrome, nausea and vomiting, alopecia, and diarrhea.

**Results:**

Eight RCTs with a total of 1998 patients with advanced gastric cancer were finally included, including 982 with capecitabine and 1016 with 5-fluorouracil. Compared with 5-fluorouracil, capecitabine use was significantly associated with an improved overall response rate in patients (RR 1.13, 95% CI 1.02–1.25, *P*=0.02). Compared with 5-fluorouracil, treatment with capecitabine was significantly associated with decreased neutropenia events (RR 0.78, 95% CI 0.62–0.99, *I*^2^ = 86%, *P*=0.04), and a decreased risk of stomatitis (RR 0.73, 95% CI 0.64–0.84, *I*^2^ = 40%, *P* < 0.0001) in patients with advanced gastric cancer. In terms of hand-foot syndrome, capecitabine was associated with increased hand-foot syndrome events than 5-fluorouracil (RR 2.00, 95% CI 1.21–3.31, *P*=0.007). In terms of thrombocytopenia, nausea and vomiting, alopecia, and diarrhea, the effect of capecitabine and 5-fluorouracil were similar (*P* > 0.05).

**Conclusions:**

Compared with 5-fluorouracil, capecitabine treatment improves the overall response rate and reduces the risk of neutropenia and stomatitis in advanced gastric cancer patients. It should be noted that capecitabine treatment may also increase the occurrence of hand-foot syndrome. Capecitabine is similar to 5-fluorouracil in causing thrombocytopenia, nausea and vomiting, alopecia, and diarrhea.

## 1. Introduction

Gastric cancer is derived from the mucosal epithelium, the vast majority of which are gastric adenocarcinomas, and more than half appear in the antrum [1]. Gastric cancer in the early stage has no obvious symptoms, or some nonspecific symptoms, such as epigastric discomfort and belching, therefore most individuals are found with advanced gastric cancer. Reference [2]. Epidemiological studies show that 0.99 million people worldwide have gastric cancer and 0.74 million people die of this disease annually [3]. There is a clear difference in incidence between males and females, with males being two to three times more prevalent than females [4]. Gastric cancer is a disease caused by both environmental and genetic factors, which is associated with genes, ethnicity, family history, geographical environment, smoking, dietary and life factors, and *Helicobacter pylori* infection [5]. At present, the treatment methods for gastric cancer include surgical treatment, chemotherapy, radiotherapy, molecular targeted therapy, and immunotherapy. The preferred way for gastric cancer is surgical treatment. However, advanced gastric cancer patients often do not have the chance of surgery [6]. Therefore, it is particularly important to select the treatment regimen for them. In recent years, molecular targeted therapy and immunotherapy have emerged endlessly, but traditional chemotherapy still plays a major role [7]. 5-Fluorouracil is the most commonly used uracil antimetabolite and is widely used to treat malignant tumors by converting to a 5-fluorodeoxyuracil nucleotide in vivo to achieve inhibition of DNA synthesis. Capecitabine, a thymidine phosphorylase active fluoropyrimidine carbamate, is a prodrug of 5-fluorouracil. Compared with 5-fluorouracil, capecitabine has no cytotoxicity. At the location of the tumor, capecitabine can be converted into 5-fluorouracil through the tumor-related vascular factor (thymidine phosphorylase), thereby minimizing the damage to normal cells. It has been shown that the capecitabine combination regimen can reduce mortality compared with 5-fluorouracil (hazard ratio (HR) 0.86, 95% confidence interval (95% CI) 0.80–0.99) [8]. However, it has also been suggested that treatment with capecitabine did not significantly prolong survival compared with 5-fluorouracil (10.5 versus 9.3 months) [9]. Therefore, the effect of capecitabine versus 5-fluorouracil remains controversial. This study aimed to investigate the effect of capecitabine versus 5-fluorouracil on overall response rate, neutropenia, thrombocytopenia, nausea and vomiting, alopecia, and diarrhea through a meta-analysis.

## 2. Materials and Methods

### 2.1. Literature Search

A literature search of PubMed, Embase, and Cochrane Library was performed to include published randomized controlled trials (RCTs) on capecitabine and 5-fluorouracil published from database establishment to June 2022. Search terms were as followed: advanced gastric cancer; advanced gastric malignancy; gastric carcinoma; gastric neoplasm; capecitabine/siroda; 5-fluorouracil; chemotherapy. The retrieved articles and references of the studies were read to try to find out the target articles as much as possible. Published clinical trials and relevant review articles in oncology journals were hand-searched.

### 2.2. Inclusion and Exclusion Criteria

Inclusion criteria were as follows: (1) RCT; (2) individuals aged ≥ 18 years with a definite diagnosis of advanced gastric cancer, despite of sex, and race; (3) capecitabine-based chemotherapy was used in the experimental group and 5-fluorouracil-based chemotherapy was used in the control group; (4) data on overall response rate and adverse events were provided in the study.

Exclusion criteria were as follows: (1) animal studies; (2) sample size < 20; (3) diagnostic criteria for advanced gastric cancer were not given; (4) the needed data was not shown, or contact authors were still not available. All searches were limited to randomized clinical trials (RCTs) reported in journals or conferences, with no publication date or language restrictions.

### 2.3. Data Extraction and Quality Assessment

Two independent researchers extracted the needed data from the included studies: first author, publication year, the sample size of both groups, patient age, patient sex, chemotherapy regimen, overall response rate, neutropenia, thrombocytopenia, stomatitis, hand-foot syndrome, nausea and vomiting, alopecia, and diarrhea. The quality was assessed by the Cochrane collaboration's risk of bias evaluation tool, including seven aspects. We also attempted to reach out to the authors for supplemental data. A third researcher will help to deal with the differences.

### 2.4. Outcome Measures

Primary outcome measures: overall response rate. Overall response rate = (complete response + partial response)/total number × 100%. Safety measures: neutropenia, thrombocytopenia, stomatitis, hand-foot syndrome, nausea and vomiting, alopecia, and diarrhea.

### 2.5. Statistical Analysis

Analysis was carried out on an intent-to-treat basis, and all randomized patients were included according to their allocated treatment. *P* < 0.05 was defined as statistically significant. RR and its 95% CI were used to analyze the effect of treatment on overall response rate, neutropenia, thrombocytopenia, stomatitis, hand-foot syndrome, nausea and vomiting, alopecia, and diarrhea in advanced gastric cancer. The *χ*2 test was used to identify the heterogeneity among RCTs. We use the fixed-effect model when *P* ≥ 0.1 and I2 ≤ 50% were considered to be small among studies; we use the random-effect model when *P* < 0.1 and *I*^2^ > 50%. Meta-analysis was performed by RevMan 5.3 software.

## 3. Results

### 3.1. Selection Process of Included Literature

**Figure 1:**
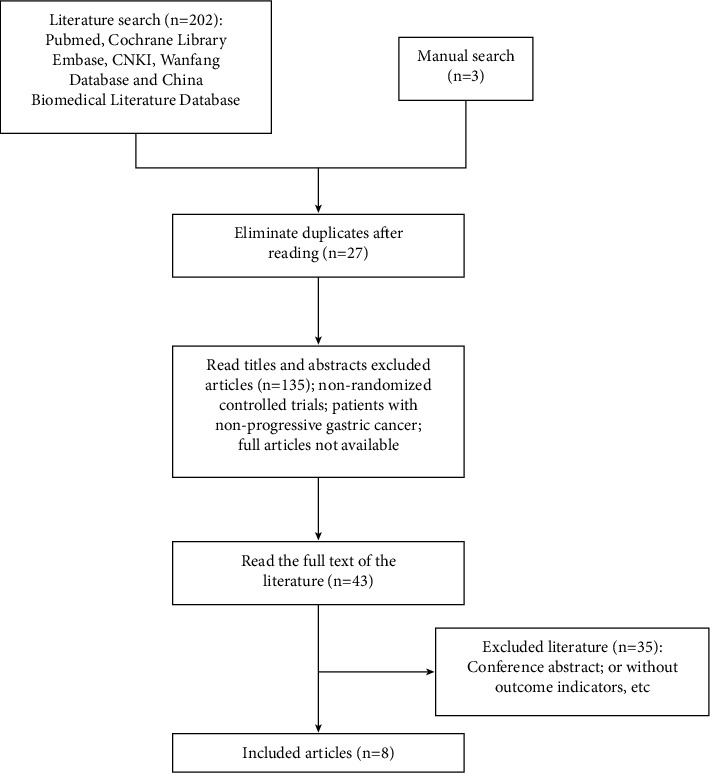
The screening process of included literature.

As shown in Figure 1, 205 pieces of screened literature were determined. We excluded twenty-seven duplicate studies and 135 studies after reading the titles and abstracts, and the remaining 43 articles were read in full. We excluded thirty-five articles with conference abstracts or without outcome measures. 8 RCTs were included in the meta-analysis [8–15].

### 3.2. Clinical Characteristics of Included Articles

1998 individuals with advanced gastric cancer were included, with 982 patients in the capecitabine arm and 1016 patients in the 5-fluorouracil arm (Table 1). The age ranged from 52 to 63 years in patients with capecitabine and from 52 to 63 years in patients with 5-fluorouracil. Both groups were predominantly male, ranging from 16 to 392 males in the capecitabine group and 13 to 393 males in the 5-fluorouracil group. Chemotherapy regimens in the capecitabine arm included cisplatin/capecitabine, docetaxel/capecitabine, and docetaxel/oxaliplatin/capecitabine. Chemotherapy regimens in the 5-fluorouracil group included irinotecan/5-fluorouracil, docetaxel/cisplatin/5-fluorouracil, and docetaxel/oxaliplatin/5-fluorouracil.

### 3.3. Risk of Bias

**Figure 2:**
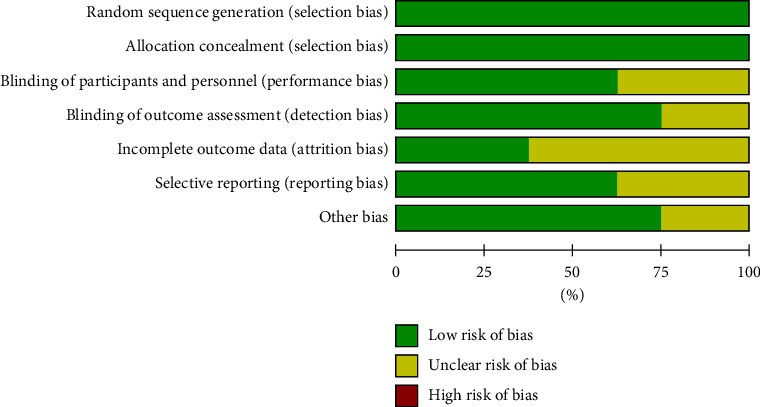
Risk of bias of included literature.

The overall study design of the included articles was good and the study quality was high (Figure 2). In terms of random sequence generation and allocation concealment, all eight studies were low risk. In terms of investigator and subject double-blinding, 5 studies were low risk and 3 were an uncertain risk. Six studies have a low risk of blinding of outcome assessment and two were an uncertain risk.

### 3.4. Effect of Capecitabine and 5-Fluorouracil on Overall Response Rate

**Figure 3:**
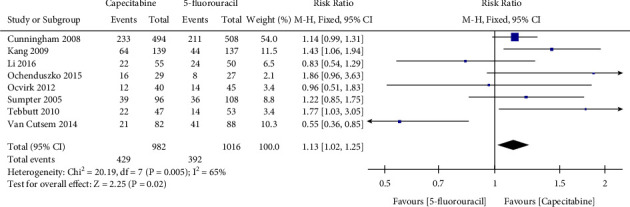
Effect of capecitabine and 5-fluorouracil on overall response rate.

A total of 8 articles were included in Figure 3 to show the effect of capecitabine and 5-fluorouracil on the overall response rate. Capecitabine use was significantly associated with an increased overall response rate compared with 5-fluorouracil (RR 1.13, 95% CI 1.02–1.25, *I*^2^ = 65%, *P*=0.02).

### 3.5. Effect of Capecitabine and 5-Fluorouracil on Neutropenia and Thrombocytopenia

**Figure 4:**
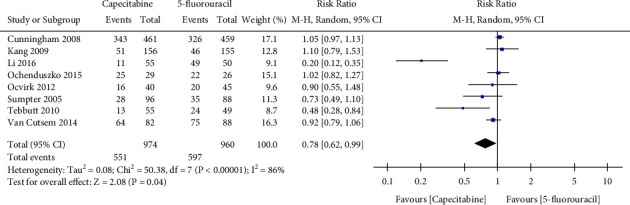
Effect of capecitabine and 5-fluorouracil on neutropenia.

Figure 4 shows the effect of capecitabine versus 5-fluorouracil on neutropenia. Compared with 5-fluorouracil, treatment with capecitabine was significantly associated with decreased neutropenia events in advanced gastric cancer patients (RR 0.78, 95% CI 0.62–0.99, *I*^2^ = 86%, *P*=0.04). Capecitabine tended to reduce the occurrence of thrombocytopenia compared with the 5-fluorouracil group (RR 0.79, 95% CI 0.38 to 1 .62, *I*^2^ = 82%, *P*=0.52) (Table 2).

## 4. Nonhematologic Adverse Events

Compared with 5-fluorouracil, the intervention with capecitabine was significantly associated with decreased stomatitis events (RR 0.73, 95% CI 0.64–0.84, *I*^2^ = 40%, *P* < 0.0001). In terms of hand-foot syndrome, capecitabine was associated with increased hand-foot syndrome events than 5-fluorouracil (RR 2.00, 95% CI 1.21–3.31, *I*^2^ = 69%, *P*=0.007). Capecitabine was not significantly different from 5-fluorouracil in nausea and vomiting (RR 0.97, 95% CI 0.91–1.03, *I*^2^ = 0%, *P*=0.27). Capecitabine did not differ significantly from 5-fluorouracil in alopecia (RR 0.95, 95% CI 0.89–1.02, *I*^2^ = 26%, *P*=0.17). Compared with 5-fluorouracil treatment, capecitabine did not significantly affect diarrhea (RR 1.02, 95% CI 0.78–1.33, *I*^2^ = 67%, *P*=0.90).

## 5. Discussion

This study found that capecitabine was significantly associated with an improved overall response rate compared with 5-fluorouracil in advanced gastric cancer individuals. In addition, capecitabine reduces the risk of neutropenia and stomatitis compared with 5-fluorouracil. However, it is noteworthy that capecitabine may increase the occurrence of hand-foot syndrome. Capecitabine is similar to 5-fluorouracil in causing thrombocytopenia, nausea and vomiting, alopecia, and diarrhea.

Over the past years, the incidence of gastric cancer has slightly decreased, which may be attributed to the improvement of people's health awareness, the progress of screening methods, and the development of molecular targeted therapy and immunotherapy [16]. However, it should be reminded that the survival rate is still low, and the five-year survival rate is still about 20% in most regions and countries of the world [17]. For advanced gastric cancer, traditional chemotherapy remains the main treatment modality. Therefore, improving disease response rate and survival rate are the main objectives when selecting chemotherapeutic agents.

A meta-analysis published in 2014, which included 26 studies with a total of 1585 patients, suggested that chemotherapy regimens containing capecitabine had similar efficacy compared with chemotherapy regimens containing 5-fluorouracil [18]. However, by further updating the literature and expanding the sample size, this study found that capecitabine may be superior in improving the overall response rate (RR 1.13, *P*=0.02). Similar to this study, capecitabine-based chemotherapy regimens have also been suggested to show longer overall survival compared with conventional 5-FU-based chemotherapy regimens [19].

In terms of safety, treatment with capecitabine was significantly associated with decreased neutropenia events (RR 0.78, *P*=0.04) and stomatitis (RR 0.73, *P* < 0.0001) compared with 5-fluorouracil. Similar to this study, an additional meta-analysis also found a significant reduction in serious leukopenia in capecitabine patients [19]. However, it is noteworthy that capecitabine may significantly increase the occurrence of hand-foot syndrome compared with 5-fluorouracil (RR 2.00, 95% CI 1.21–3.31 *P*=0.007). Grade 1 or 2 hand-foot syndrome can be managed by ointment or appropriate reduction [20]. Some studies suggest that the hands and feet have many exocrine glands, and the excretion of capecitabine from the suboriferous is the cause of the hand-foot syndrome. In addition, the hand-foot syndrome caused by capecitabine may be related to the destruction of the deep capillary network and the overexpression of cyclooxygenase (COX-2).

The limitations of the study were as follows: (1) the primary endpoint evaluated in this study was the overall response rate without assessing survival. Although the overall response rate is clearly associated with survival, survival is the more important indicator. Future studies are needed to further evaluate survival. (2) Adverse reactions such as neutropenia, thrombocytopenia, nausea and vomiting, alopecia, stomatitis, and diarrhea were assessed in this study, but safety events such as anemia and peripheral neuropathy were not assessed. (3) Although the chemotherapy treatments included in the study are based on capecitabine or 5-fluorouracil, there are some differences between the chemotherapy plans in different studies, which may affect the results of the study. (4) Meta-analysis is mainly used to identify the relevant relationship, and a large sample of head-to-head *R* CT is needed in the future to further compare the effect of capecitabine and 5-fluorouracil.

In summary, capecitabine treatment improves overall response rates and reduces the risk of neutropenia and stomatitis compared with 5-fluorouracil. It should be noted that treatment with capecitabine may also increase the occurrence of hand-foot syndrome. Capecitabine is similar to 5-fluorouracil in causing thrombocytopenia, nausea and vomiting, alopecia, and diarrhea.

## Figures and Tables

**Table 1:** Clinical characteristics of included literature.

First author	Year	Sample size (capecitabine/5-fluorouracil)	Age, years (capecitabine/5-fluorouracil)	Number of males (capecitabine/5-fluorouracil)	Capecitabine arm chemotherapy regimen	5-Fluorouracil arm chemotherapy regimen
Cunningham [8]	2008	494/508	63/63	392/393	Epirubicin/cisplatin/capecitabine, or epirubicin/oxaliplatin/capecitabine	Epirubicin/cisplatin/5-fluorouracil, or epirubicin/oxaliplatin/5-fluorouracil
Kang [9]	2009	139/137	56/56	103/108	Cisplatin/capecitabine	Cisplatin/5-fluorouracil
Li [10]	2016	55/50	52/52	35/34	Epirubicin/oxaliplatin/capecitabine	Irinotecan/5-fluorouracil/leucovorin calcium
Ochenduszko [11]	2015	29/27	58/60	16/13	Epirubicin/oxaliplatin/capecitabine	Docetaxel/cisplatin/5-fluorouracil
Ocvirk [12]	2012	40/45	56/55	32/34	Epirubicin/cisplatin/capecitabine	Epirubicin/cisplatin/5-fluorouracil
Sumpter [13]	2005	96/108	63/62	82/79	Epirubicin/cisplatin/capecitabine, or epirubicin/oxaliplatin/capecitabine	Epirubicin/cisplatin/5-fluorouracil, or epirubicin/oxaliplatin/5-fluorouracil
Tebbutt [14]	2010	47/53	59/61	42/42	Docetaxel/capecitabine	Docetaxel/cisplatin/5-fluorouracil
Van Cutsem [15]	2014	82/88	59/58	74/69	Docetaxel/oxaliplatin/capecitabine	Docetaxel/oxaliplatin/5-fluorouracil

**Table 2:** Safety analysis of capecitabine and 5-fluorouracil.

Adverse reactions	Capecitabine arm (number of events/sample size)	5-Fluorouracil group (# events/sample size)	RR	95% CI	*P* Value	*I* ^2^ (%)
Thrombocytopenia	130/811	146/813	0.79	0.38–1.62	0.52	82
Nausea and vomiting	580/969	598/969	0.97	0.91–1.03	0.27	0
Hand-foot syndrome	300/878	173/872	2.00	1.21–3.31	0.007	69
Alopecia	454/689	477/693	0.95	0.89–1.02	0.17	26
Stomatitis	238/878	322/872	0.73	0.64–0.84	<0.0001	40
Diarrhea	372/878	364/872	1.02	0.78–1.33	0.90	67

## Data Availability

The analyzed data sets generated during the study are available from the corresponding author upon reasonable request.
